# An Improved Vital Signal Extraction Method Based on Laser Doppler Effect

**DOI:** 10.3390/s24217027

**Published:** 2024-10-31

**Authors:** Yu Li, Haiyang Zhang, Bowen Zhang, Yujiao Qi, Si Chen

**Affiliations:** School of Optics and Photonics, Beijing Institute of Technology, Beijing 100081, China

**Keywords:** Laser-Doppler, vital signal, signal filtering, waveform decoupling

## Abstract

The mixed signal of respiratory waveform and heartbeat waveform detected by the Laser-Doppler system is processed with an intermediate-frequency (IF) interference filtering method, an enhanced extraction method and a waveform-fixing method. To filter the IF interference signals and the noise scatters in the time-frequency graph, the filtering method based on coefficient of variation (CoV) values and the enhanced curve extraction method based on noise-scatter theory are utilized in vital signal analysis. To decouple the respiratory signal and the heartbeat signal in time domain, the waveform-fixing method based on second-order difference theory is utilized in signal decoupling. This method as an algorithm is applied in the computer simulation and laboratory environments. The results show that the above methods can extract the mixed waveforms and identify the respiratory rates and heart rates in real experimental data. The IF interference signal can be filtered adaptively, and the accuracy of the analyzed rates can be improved to about 95%.

## 1. Introduction

The monitoring of the respiration and heartbeat of a patient is typically required in medical diagnosis, and it indicates the patient’s health status and provides significant guidance to medical staff for deciding about any further treatment that the patient may require. The equipment currently being used to monitor respiratory and heartbeat signals of patients in the intensive care units of hospitals is usually attached to the patients using patches or tapes. These patches/tapes can cause discomfort to the patients and can even cause allergic reactions in them, leading to the spread of the COVID-19 disease. Therefore, a noncontact method is required for measuring the vital signals of the human body.

Because the respiration and heartbeat motions will cause a human body surface to vibrate, we can apply the laser coherence method utilized in the industry [1,2] to measure the body surface vibrations of a person and then demodulate the reflected wave to obtain the respiratory and heart rates of the person. Related studies used this method to measure chest wall [3,4,5] or carotid artery [6,7] vibrations to test the effectiveness of the theory. Some studies [8,9] have demonstrated the feasibility of indirect vital-signal measuring from multiple orientations, including the back, indicating that the human body’s surface contains much information about respiration and heartbeat. However, the signal-to-noise ratio (SNR) of reflected waves determines the quality of analysis results, and the above works used high reflectivity tapes or liquid to improve the SNR, which still made it inconvenient for the patient. In addition, the vibration of the chest is a mixed movement caused by both respiration and heartbeat. Although several related studies used a filter to decouple the two signals [10], some studies proved this method may become unreliable, or even wrong in some cases [11], or consider the vital signals as overly idealized analytical functions (such as tangent function [12]), which does not adequately account for real-human conditions. Therefore, a variety of unconventional waveform and spectral processing methods have been proposed. More direct methods, for example, differential enhancement (DE), eliminate the effects of the respiration harmonic interference on HR estimation [13]; a “variable-length” wavelet transform method has also been used to process these irregular vital signals [14]. Other studies have used methods based on empirical mode decomposition (EMD) to decouple the mixed signals into many intrinsic mode functions (IMFs), and several IMFs will be chosen as target signals [15], but how to choose correct IMFs is a tough problem.

Additionally, there are significantly more systems and measurement methods based on millimeter-wave radar, compared to those based on laser heterodyne interferometry. Techniques commonly used such as I-Q demodulation and range-Doppler mapping cannot be directly applied to the analysis of laser heterodyne interferometric signals [16,17,18,19,20].

In this paper, we present new algorithms used in a laser heterodyne interferometric system to decouple the respiratory and heartbeat signals, including an IF interference signal filtering algorithm based on CoV, a curve extraction method in extremely low SNR cases based on noise-scatter theory and a waveform-fixing method based on the signal-difference and period-average. The proposed method used simple and feasible logic to filter the IF interference adaptively, extract the centroid curve from graph with low SNR and optimize the two signal waveforms. The system will have fewer requirements compared to other systems, and a new concept of signal decoupling is introduced.

## 2. Signal Processing Theory and Simulation Results

### 2.1. System Structure and Formula Derivation

**Figure 1:**
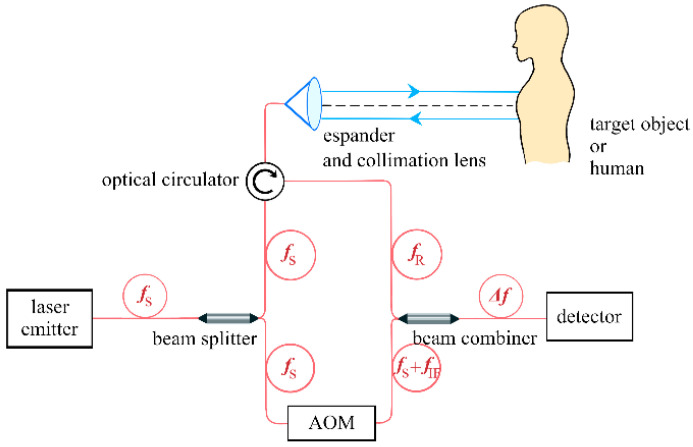
Schematic diagram of the Doppler-LIDAR system. When the vibrating object is a human being, the laser beam should be aimed at the chest of the person. AOM stands for Acousto-Optic Modulator.

The Doppler-LIDAR system has been designed to transmit a laser of known frequency and detect the frequency of the reflected laser. The schematic diagram of the system is shown in Figure 1.

If we consider the frequency of the transmitted laser $\;f_{s}$, velocity of the targets $v_{ob}$, speed of light in the medium where the laser source and the target are located u and the frequency of the laser reflected from the target $f_{R}$, then considering the Doppler effect, the relationship between the frequencies of the incident and reflected lasers can be expressed using Equation (1) below.

(1) $$\begin{array}{c} f_{ob}=\frac{u+v_{ob}}{u}f_{s}\; \end{array}$$

Due to the Doppler effect, the frequency of the laser detected by the system, $f_{R}$, will be different from $f_{ob}$, and it can be expressed as follows:

(2) $$\begin{array}{c} f_{R}=\frac{u}{u-v_{ob}}f_{ob} \end{array}$$

However, since the speed of laser u is light-speed c, the Laser-Doppler effect must account for time dilation. Therefore, the frequency of the echo can be expressed as:

$$f_{R}=\frac{1+\frac{v_{ob}}{c}}{1-\frac{v_{ob}}{c}}f_{s}$$

Considering that the velocity of the object is much smaller than light-speed, a first-order Taylor expansion (Maclaurin series) can be used, leading to:

$$f_{R}=\left(1+\frac{2v_{ob}}{c}\right)f_{s}$$

In the heterodyne coherence method, we typically make the frequency of the reference laser higher or lower than the frequency of the transmitted laser by a specified amount. If the difference between the two frequencies is $f_{IF}$, then the total difference between the frequencies of the reflected and reference lasers can be represented as

(3) $$\begin{array}{c} \Delta f=f_{IF}+\frac{2v_{ob}}{c}f_{s} \end{array}$$

Thus, using Δf, the velocity of the target can be expressed as

(4) $$\begin{array}{c} v_{ob}=\frac{\lambda }{2}\left(\Delta f-f_{IF}\right) \end{array}$$

Thus, we need to accurately extract and record the frequency of the reflected signal, especially when it can change with time. By analysing the velocity of the target across time, we can obtain the movement statement of the target. If the velocity is in cm∕s, the wavelength of the laser is in μm, then the inspected frequency should be approximately in $\mathrm{the}\;\mathrm{order}\;\mathrm{of}\;10^{4}$ Hz.

We considered the vibration of a human chest wall to be due to be caused by both respiration and heartbeat. The respiratory and heartbeat signals can be considered periodic signals with two different periods. The amplitude of the respiratory signal is larger than that of the heartbeat signal (“amplitude” means the movement of the chest wall caused by respiration or heartbeat). If we use sinusoidal signals of different frequencies and amplitudes to represent the respiratory and heartbeat signals, then the combined signal *Com*(*t*) can be expressed as

(5) $$Com\left(t\right)=res\left(t\right)+hea\left(t\right)\;=A_{r}\sin\left(2\pi f_{r}t\right)+A_{h}\sin\left(2\pi f_{h}t\right)\;$$

where $A_{r}$ is the amplitude of the respiratory signal, $A_{h}$ is the amplitude of the heartbeat signal, $f_{r}$ is the frequency of the respiratory signal and $f_{h}$ is the frequency of the heartbeat signal. A sinusoidal signal will not accurately represent the form of the combined signal, but it will be able to show the characteristics of the two signals. Thus, we can express the velocity of the chest wall as follows:

(6) $$Vel\left(t\right)=\frac{dCom\left(t\right)}{dt}\;=A_{r}\cdot 2\pi f_{r}\cdot \cos\left(2\pi f_{r}t\right)+A_{h}\cdot 2\pi f_{h}\cdot \cos\left(2\pi f_{h}t\right)\;=B_{r}\cos\left(2\pi f_{r}t\right)+B_{h}\cos\left(2\pi f_{h}t\right)$$

Thus, the velocity signal can be regarded as a combination of two sinusoidal signals with a phase shift.

### 2.2. Algorithm Theories and Simulation Results

**Figure 2:**
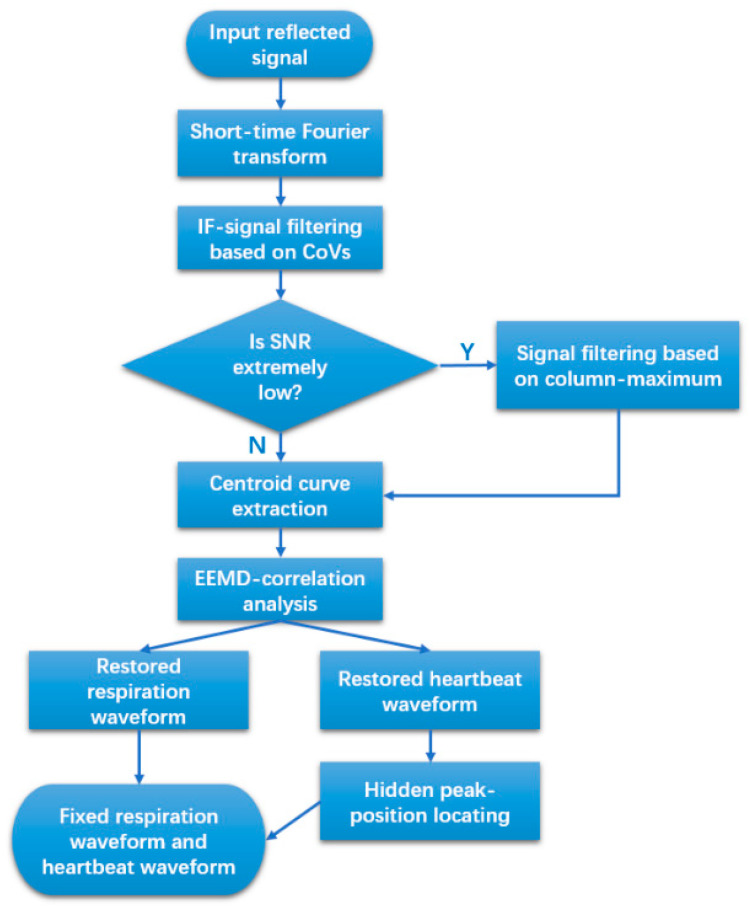
Flowchart of the algorithm.

The flowchart of the algorithm of the proposed method, which mainly contains two new filtering methods and a new waveform restoration method, is shown in Figure 2. The reflected signal will be uploaded to a personal computer and transformed into time series data. The time series data will be analyzed using the short-time Fourier transform (STFT) method and filtered using the new algorithm based on the threshold of CoV. The system will thereafter check whether the SNR of the signal is high enough to enable the extraction of the centroid curve. If the SNR of the signal is much lower than the stipulated minimum SNR, then the system will perform further filtering based on the maximum of each time unit. After the centroid curve has been extracted, the ensemble empirical mode decomposition (EEMD)-correlation will be performed to obtain two preliminarily restored waveforms. Then, using the waveform-fixing algorithm and based on the hidden peak positions of the heartbeat waveform, we can obtain the respiration waveform.

**Figure 3:**
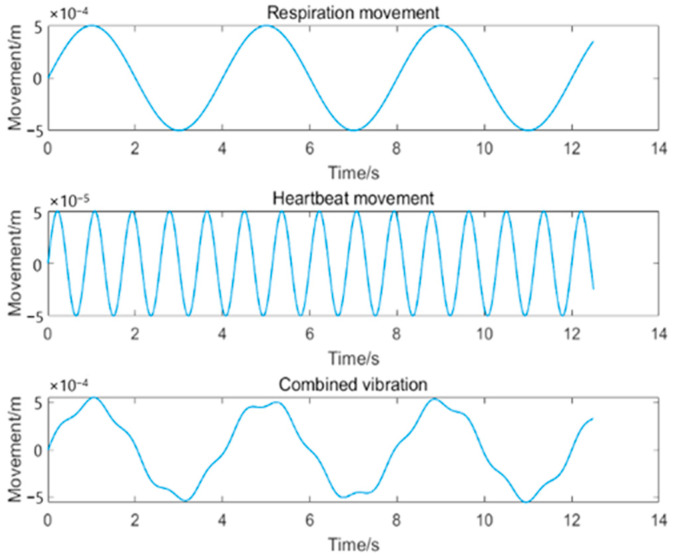
Simulated respiration and heartbeat waveforms.

We can generate the simulated vibration signal as shown in Figure 3. Based on the reference respiratory and heartbeat rates and amplitudes used in related studies [21,22], we can consider the heart rate as 70 per minute and respiratory rate as 15 per minute, and their amplitudes as 0.05 and 0.5 mm, respectively. The amplitude of the vibration signal due to the heartbeat is the displacement of the chest wall caused by the heartbeat and not by the heart itself.

**Figure 4:**
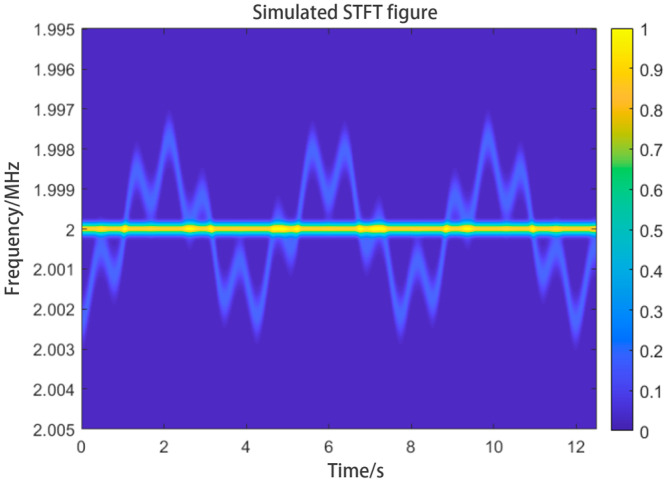
Simulated STFT plot showing IF interference.

In Equation (3), if we set $f_{IF}$ to 2 MHz, $f_{s}$ to $3\times 10^{14}$ Hz and *u* to $3\times 10^{8}$ m/s, the STFT plot of the signal obtained will contain the patterns of Δf and $f_{IF}$ as shown in Figure 4. The graph obtained using the STFT method will have time on the horizontal axis and frequency on the vertical axis. The graph will show the variation of the signal frequency with time. We can call this graph the “time–frequency graph”. We can analyze and extract information from the graph to obtain the velocity of the target. In practical applications, because of the reflectivity of the target and settings of the optical components, the time–frequency graph can contain an extremely bright lateral interference at $f_{IF}$. We call this IF interference. The simulated graph shows the IF interference.

#### 2.2.1. IF Interference Signal Filtering Based on Coefficient of Variation

**Figure 5:**
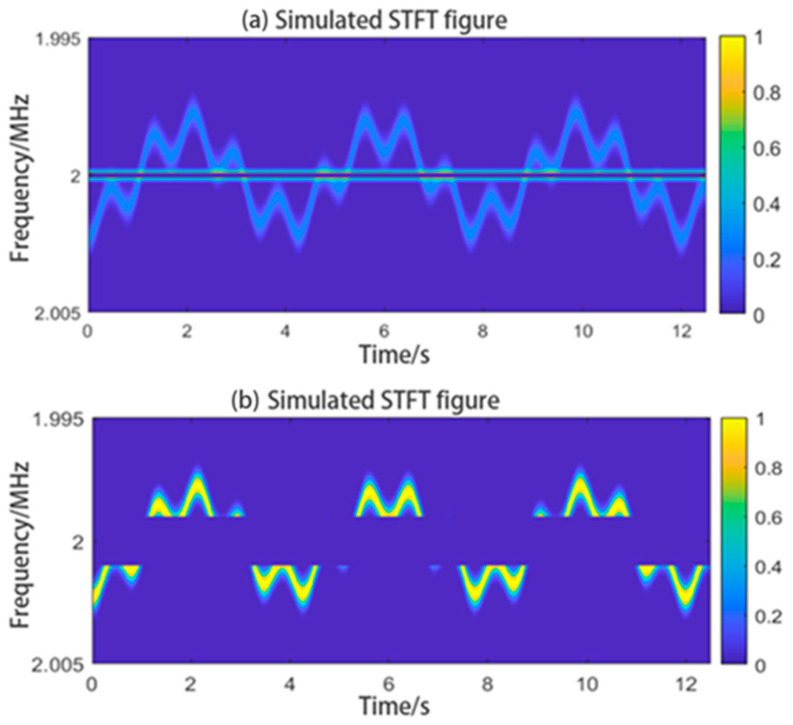
STFT plot obtained after filtering the signal using filters with different pre-set frequency ranges. If the range is too narrow, residual IF interference would remain, and if the frequency range is too wide, even part of the original signal would be filtered out. Because the image intensity has been normalized, the two images have different colors.

We can consider the waveform shown in Figure 4 as a mixture of the waveforms of the intermediate frequency and velocity signals, and extract the required waveforms from the velocity signal waveform. Related studies [23] used the centroid curve extracting method to obtain curve data from images. The method we used can extract only one waveform from the waveform shown in Figure 4, and we need to first filter out the intermediate-frequency waveform from it. According to most of the related studies, using a filter with a preset-frequency range, we can remove the signals falling within that range. We chose to filter out signals falling within two different preset-frequency ranges, and the results are shown in Figure 5. We can see that if the frequency range is not correctly chosen, there will be residual IF interference, or the signal would be filtered to such an extent to affect the extraction of the centroid curve. In a worse situation, there could be more than one IF interference signal, making it impossible to predict the frequency ranges of the filters.

In this paper, a method is proposed for filtering the IF interference signal based on CoV. We assumed that the IF interference contained in the time–frequency graph has geometric characteristics, that is, the IF interference will usually fall within a certain frequency range, and its power will be relatively constant. Therefore, we adopted the concept of “CoV used in mathematics”. The CoV of a data series can be defined as

(7) $$\begin{array}{c} C_{v}=\frac{\sigma }{\mu } \end{array}$$

where σ is the standard deviation of the data series and μ is the average value of the data series. The CoV measures the fluctuation of the data and eliminates the influence of its own average value, which standard deviation cannot show. The CoVs of different series of data can be directly compared.

**Figure 6:**
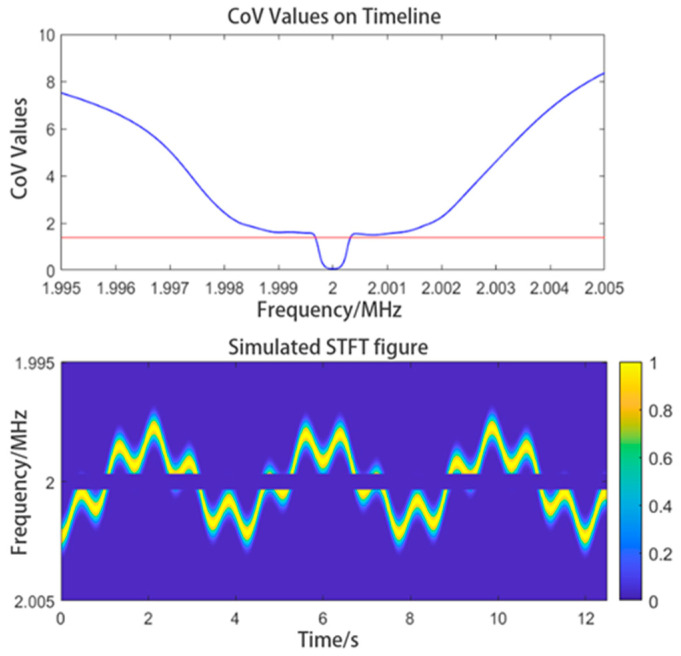
CoV distribution and the time-frequency graph obtained after filtering the IF interference. In the image of CoV values, the red straight line indicates the threshold, and the blue curve indicates the CoV values.

The fluctuation of the IF interference is small, and its CoV is lower than those in other frequency ranges. Thus, IF interference can be accurately determined by calculating the CoV of each row along the time axis, then, as shown in Figure 6, we would be able to obtain the graph showing the distribution of the CoV over the frequency range considered.

In Figure 6, the blue curve represents the CoV distribution, and the red line represents the CoV threshold selected. We can see many valleys near the 0-amplitude point in the CoV distribution, which correspond to the frequency of the IF interference. The CoV distribution at its outermost end has to be ignored because it provides no valid information. If the threshold of the CoV has been appropriately set, the signals in the frequency range within which the IF interference lies will be all filtered out, while those whose frequencies are outside that frequency range will be retained. This method can be used to filter out IF interference adaptively; it could also be used to filter out discretely distributed IF interferences. The method would not be significantly affected by the intensity of the IF interference. We can obtain the time–frequency graph of the signal after the IF interference has been filtered out, as shown in Figure 6.

It should be noted that the threshold needs to be manually selected for now and may vary with changes in signal quality. Typically, it is necessary to collect a segment of the signal under the same hardware conditions, then adjust the threshold multiple times to test the filtering effect and determine the optimal threshold.

#### 2.2.2. Centroid Curve Extraction Method for Use with Signals with Extremely Low SNRs

**Figure 7:**
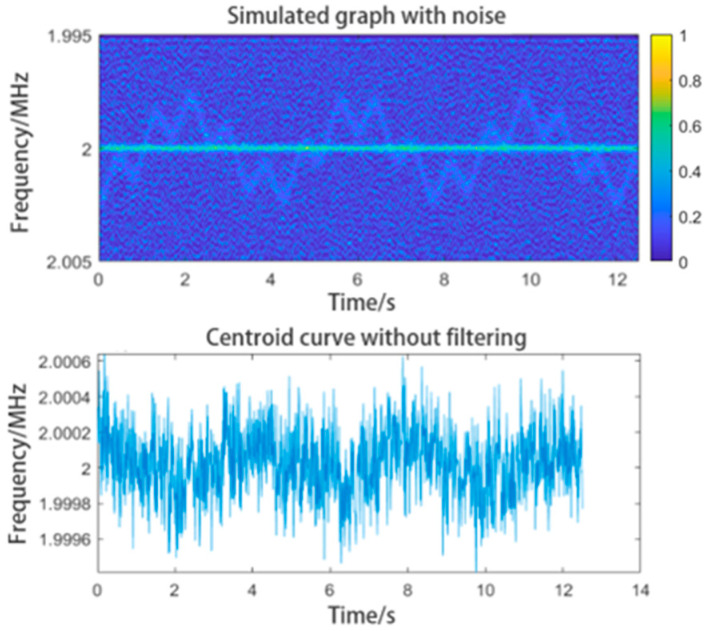
Time-frequency graph containing noise and the centroid curve extracted directly from the graph.

In related studies, the time–frequency graph has often been converted into a frequency–time curve using the centroid extraction method. However, in practical applications, because of the low SNR of the reflected signal, the graph may include considerable noise. We noticed random noise in the graph caused by the low reflectivity of the target. This low reflectivity submerges the highest intensity peak in the spectrum of the reflected signal in noise. Even the valid information can become intermittent. We added simulated noise to the graph, as shown in Figure 7.

When the quality of the graph is low, the centroid curve extracted from the graph will contain noise and even errors. This will reduce the accuracy of data processing. The centroid curve extracted from this graph is shown in Figure 7, which contains too much noise and its shape is unacceptable.

The extraction of the centroid curve from the graph directly using image-processing methods will not be appropriate because of the following two reasons:

1. Considerable difference exists between the horizontal and vertical axes units (in the graph, the frequency axis is divided into 25,001 units, whereas the time axis is divided only into 625 units). The width of the valid information lies within a narrow time range, and thus the extraction of the centroid curve using a high-pass filter would be difficult.
2. When the graph is converted into an image using a specific scale, some details may be discarded owing to the image quality of the display.

In this paper, we propose a centroid curve extraction method for use with signals with extremely low SNRs.

**Figure 8:**
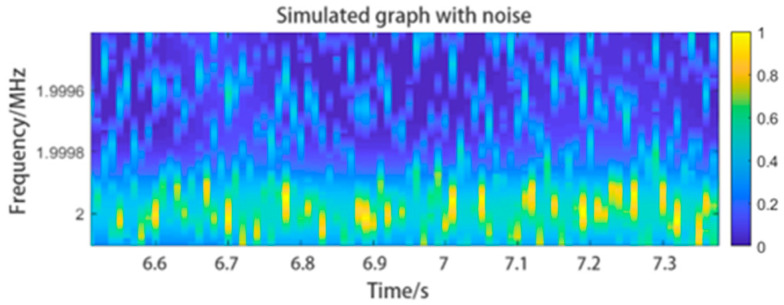
Scatters present in the graph. Some of the scatters relate to valid information, while others relate to noise.

Preliminary filtering is first done to filter out the IF interference, then, several units with the largest power in each column are selected to obtain a clear graph, which will contain considerable noise. We can consider the noise points in the graph as “scatters”. The “scatter” is defined as the set of pixels between each pair of consecutive local minima in the intensity values of a column in the time–frequency graph. In essence, the scatter in one column corresponds to the individual bumps in the spectrum at this moment. So, the span of each scatter has several units on the frequency axis, with only one unit on the time axis. It is evident that the valid information corresponds to the main peak in the spectrum, and the corresponding scatter will be prominent. The scatters corresponding to noise will be distributed in other regions and will have weaker intensity. Some of the scatters are shown in Figure 8.

The key to filtering this graph is to distinguish between scatters relating to valid information and those relating to noise. Based on the difference among the lengths and intensities of the scatters, we developed a test algorithm to count the length and intensity of each scatter, and set the length and intensity thresholds.

**Figure 9:**
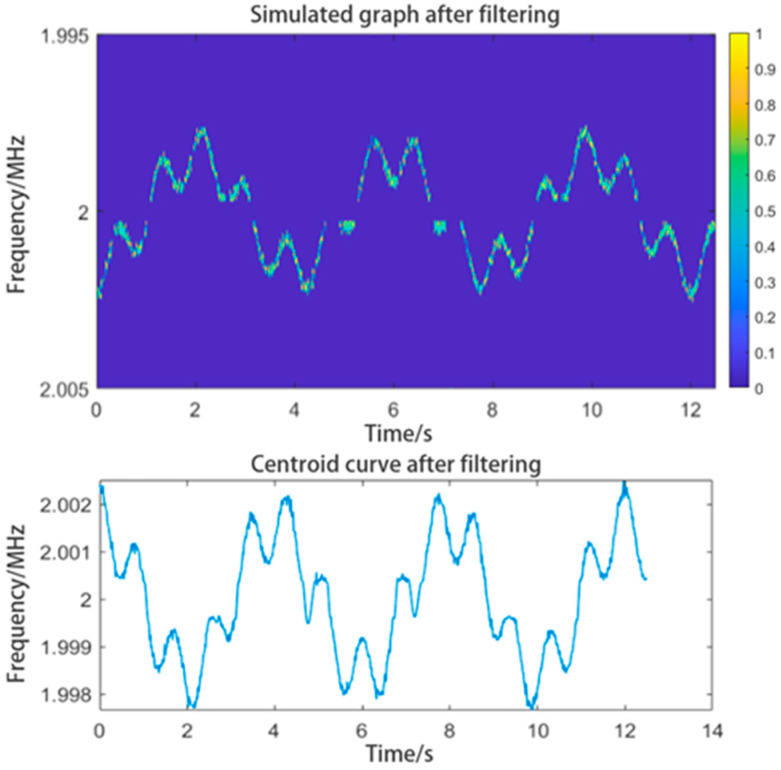
The graph after filtering and the centroid curve extracted from the filtered graph. The curve has a clear shape.

Scatters are segmented based on the positions of local minima in each column, with each segment between two consecutive local minima being considered as a single scatter. The lengths of all scatters in each column are statistically analyzed to obtain the distribution of scatter lengths. Simultaneously, the total intensities of all scatters in each column are also statistically analyzed to obtain the distribution of scatter intensities. By setting a threshold ratio (typically small, such as 10%), scatters that rank in the top 10% in both length and intensity are considered valid information, while the remaining scatters are removed as noise. This process is applied to each column to achieve filtering. The graph after being processed is shown in Figure 9. In the Figure, the valid information has become clear.

Using the same centroid curve extraction method, we can obtain the centroid curve shown in Figure 9. By adjusting the relevant parameters, the centroid curve could be restored. Although the curve may contain fluctuations, it will retain the high-frequency components.

#### 2.2.3. Waveform-Fixing Method Based on the Difference and Period-Average Values

**Figure 10:**
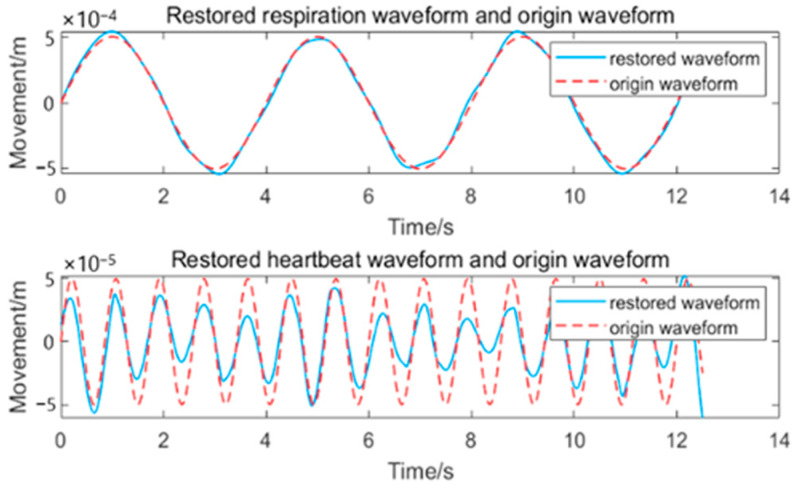
Simulatedheartbeat-andrespiratorysignalwaveforms extactedfrom the centroid curve.

After integrating the centroid curve and converting the units based on Equation (4), we can obtain the movement curve, which is a mixture of the respiratory and heartbeat waveforms. For the mixed signal, some related studies typically used the EEMD-correlation analysis to decouple the mixed waveform and obtain the IMFs as respiration and heartbeat waveforms [7,10]. This method makes high demands on the SNR of the signal to make it produce acceptable results. When the signal is decoupled using the method mentioned, we obtain the respiration and heartbeat signals. The results are shown in Figure 10.

Although the respiratory and heartbeat waveforms have been well restored, some peaks in the restored heartbeat waveform have deviated from those that were present in the original heartbeat waveform, leading to the distortion of the respiratory waveform. In practical applications, several other displacements with higher or lower frequencies will be present in the mixed waveform, which may submerge the peaks in the heartbeat waveform.

**Figure 11:**
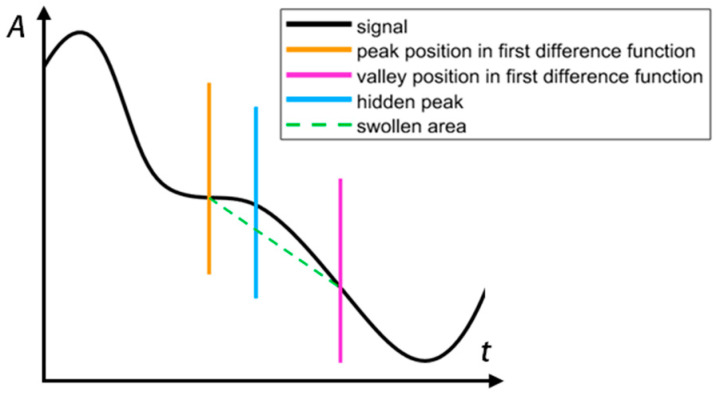
The method to mark the hidden peaks.

In this paper, we propose a waveform correction method based on the signal-difference and period-average values. First, we calculate the first difference function of the heartbeat waveform. According to mathematical principles, between the peak and valley of the first difference function of the original heartbeat waveform, a swell will be present, as shown in Figure 11. The orange line in the figure marks the position of the peak in the first difference function, while the purple line marks the position of the valley in the function.

**Figure 12:**
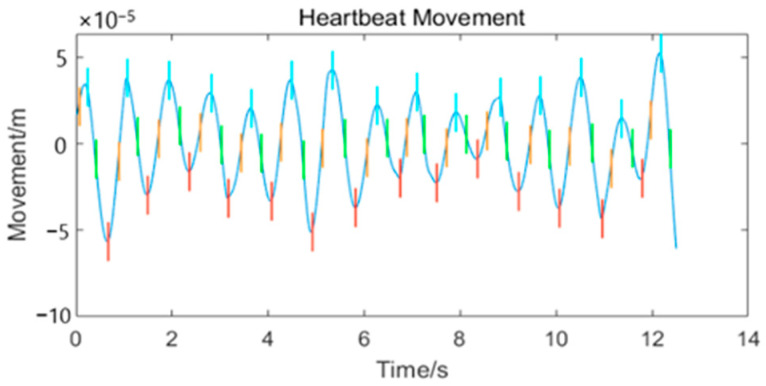
Peaks and valleys of the heartbeat waveform. In this image, the cyan line represents the peak positions, the red line represents the valley positions, and the orange and green lines represent the extremum positions of the first-order difference of the curve.

The swell between the orange and purple lines indicates a hidden peak at the position of the swell. The green dotted line demarcates the swollen area. The point at which the largest difference between the green dotted line and the waveform above it exits is marked using the blue line. We can assume that the blue line marks the exact location of the hidden peak. It is easy to notice that the exposed peaks can also be marked, so this method can be used to mark the positions of all peaks in the waveform. We can similarly mark the valley positions using a red line, as shown in Figure 12 (because the waveform has been restored well, no hidden peaks have to be added. More comparison is in Section 3).

Once we have marked its peak and valley positions, the respiratory waveform can be restored to a certain extent. The original mixed waveform Com(t) can be cut into N segments based on peak-positions of heartbeat waveforms. Then,

(8) $$Com\left(t\right)=res\left(t\right)+hea\left(t\right)\;\;=\{\begin{array}{c} res_{1}\left(t\right)+hea_{1}\left(t\right),t\in T_{1}\\ res_{2}\left(t\right)+hea_{2}\left(t\right),t\in T_{2}\\ \ldots \\ res_{N}\left(t\right)+hea_{N}\left(t\right),t\in T_{N} \end{array}$$

Define time interval for segment i to be $T_{i}=\left[t_{i-1},t_{i}\right],i=1,2,\ldots ,N$. It is noteworthy that $t_{0}$ and $t_{N}$ do not necessarily correspond to the start and end of the data acquisition period. If we assume that the heartbeat waveform has a regular form in each segment, and that its periodicity is stable, then we could assume that the integral value of each segment of hea(t) is a constant $C_{h}$, calculated as indicated below.

(9) $$average_{i}=\frac{\sum_{t\in T_{i}}\left(res\left(t\right)+hea_{i}\left(t\right)\right)}{length\left(T_{i}\right)}\;\begin{array}{c} =\frac{\sum_{t\in T_{i}}res\left(t\right)}{length\left(T_{i}\right)}+\frac{hea_{i}\left(t\right)}{length\left(T_{i}\right)}\\ =\bar{res\left(t\right)}+\bar{hea\left(t_{i}\right)}\\ ={\bar{res\left(t\right)}}_{t\in T_{i}}+C_{h} \end{array}$$

${\bar{res\left(t\right)}}_{t\in T_{i}}$ represents the average value of res(t) in segment $T_{i}$. We can calculate the average value of each segment of Com(t) using the following equation:

(10) $$average=\{\begin{array}{c} \begin{array}{c} {\bar{res\left(t\right)}}_{t\in T_{1}}+C_{h}\\ {\bar{res\left(t\right)}}_{t\in T_{2}}+C_{h} \end{array}\\ \begin{array}{c} \ldots \\ {\bar{res\left(t\right)}}_{t\in T_{N}}+C_{h} \end{array} \end{array}\;\;=res\left(T_{i}\right)+C_{h}$$

**Figure 13:**
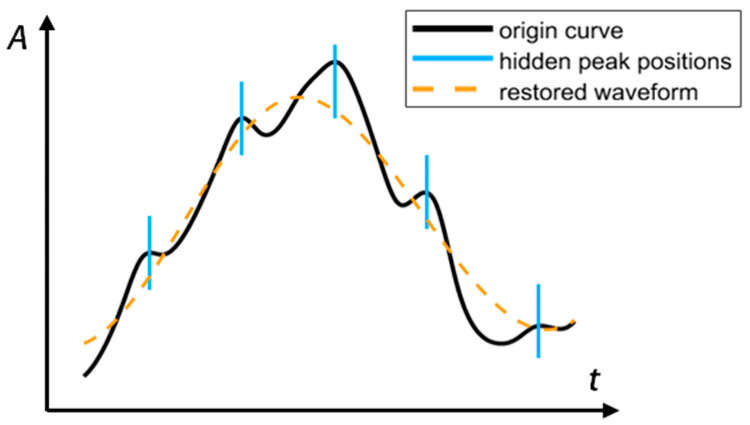
Restoration of the respiratory waveform.

**Figure 14:**
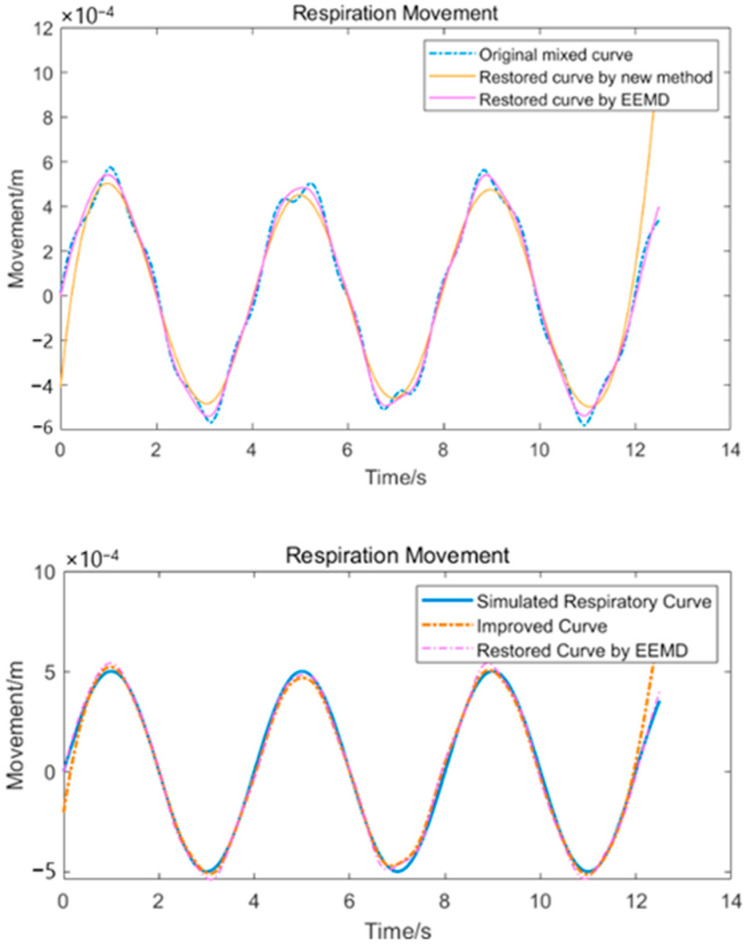
Original mixed waveform and the two respiratory waveforms obtained using different methods.

**Figure 15:**
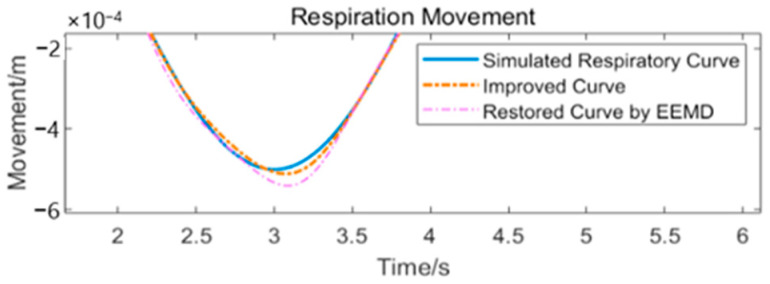

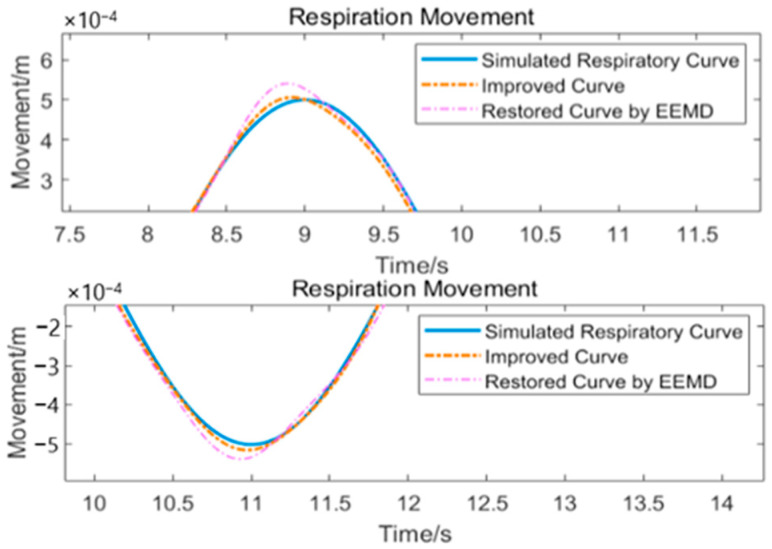
Details of the improved and original waveforms.

In Equation (10), $res\left(T_{i}\right)$ represents the discrete form of res(t), showing the discrete sampling distribution over each segment. The processing can be shown as in Figure 13. Equation (8) shows that the scatter sequence we obtained consists of two parts: one is the data from the respiratory waveform bre(t), and the other is the constant offset $C_{h}$. Because the addition of a constant offset to the waveform will not affect its shape, we can use the shape of the waveform obtained using the average value as a reference. Because smoothing of the waveform using interpolation will introduce distortion, we can combine the waveform recovered by EEMD and the waveform obtained by the method described above. Figure 14 shows the original mixed waveform and the respiratory waveforms obtained using different methods, and Figure 15 shows the improved combined waveform. The improved respiratory waveform has several similarities to its original waveform.

We can clearly see that the restored respiratory waveform has been modified in many places and that it closely resembles the original simulated respiratory waveform. The details are shown in Figure 15. This method does not depend on an analytical expression or a pre-set shape for waveform fitting, and has the potential to reduce model mixing.

## 3. Experimental Results and Their Analysis

**Figure 16:**
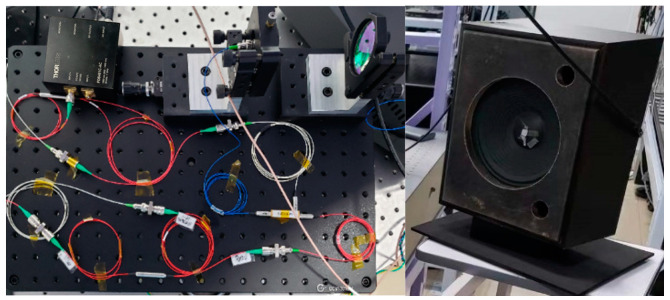
Experimental devices.

We used the devices shown in Figure 16. The emitted laser will reach the vibrating object to be reflected back to the detector through the lens at the top-right corner. Once the detector receives the coherent signal, it will output a voltage signal to a personal computer to be processed using the proposed algorithms. The algorithms will be tested using the experimental device shown in Figure 16.

In the experiment, the wavelength of laser was 1064 nm. The sampling rate was set to 250 MHz to match the 100 MHz acousto-optic frequency shift.

The experimental verification of the algorithms presented in Section 2 was performed in a laboratory environment to obtain and analyze the signal in low SNR conditions and obtain the restored waveform. The loudspeaker box and human chest wall were considered as the vibrating objects in the experiments.

### 3.1. Measurements Made on the Loudspeaker Box

**Figure 17:**
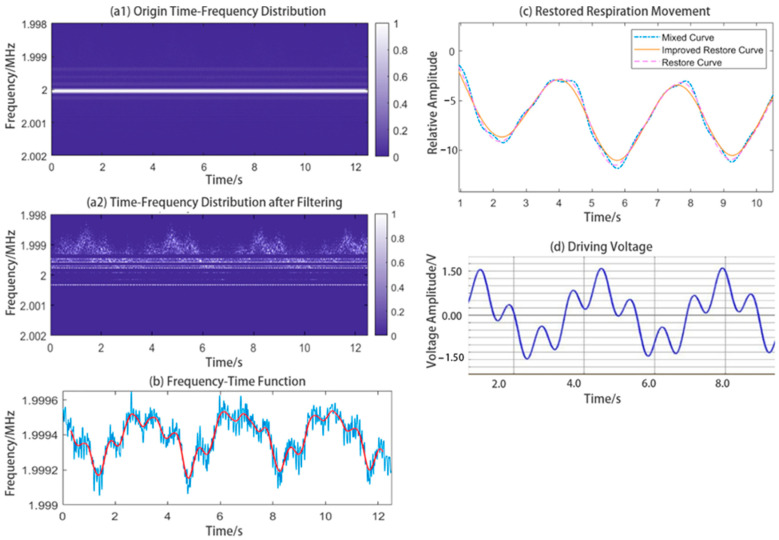
Experimental results obtained for the loudspeaker box, including the filtered time-frequency graph, centroid curve, and restored respiratory curve. Driving voltage of the loudspeaker box is shown in the last subfigure. The vibration of the loudspeaker box is proportional to the driving voltage. (In (**b**), the blue line represents the origin centroid curve, and the red line represents the curve after simple filtering).

We fed a voltage signal to the loudspeaker box to generate a vibration containing two sinusoidal waveforms with two different frequencies and amplitudes. The two pre-set frequencies were 17 and 70 bpm. The loudspeaker box is shown in Figure 16. The voltage signal tested on the loudspeaker box is shown in Figure 17d.

The graph and curve being processed are also shown in Figure 17. First, we obtained the origin time–frequency graph with strong IF interference, as shown in Figure 17a1, which made the valid information nearly undetectable in the normalized view. Then the time–frequency graph was filtered by a method based on CoVs to remove the IF interference, as shown in Figure 17a2. The centroid curve was extracted from the graph. By removing the IF interference accurately, the graph can be processed better. The centroid curve extracted and smoothed from the time–frequency graph is shown in Figure 17b. We can see that the centorid curve shows clear geometry features. It is interfered with by the IF interference as little as possible. After decoupling and waveform-fixing, we can obtain the low-frequency waveform as the respiratory waveform, as well as the fixed respiratory waveform. These waveforms are shown in Figure 17c.

The experimental results of the frequency of respiratory and heart rate are 14.8 and 69.0 bpm, and the relative errors are 13% and 1.4%, respectively. The improved restored curve shows similarities to the original waveform of the voltage fed to the loudspeaker box (Figure 17d). The accuracy of the high-frequency waveform has improved, and the frequency of the low-frequency waveform contains an increased number of errors owing to the edge effect. We think this may be related to the length of time of data, and, as time goes on, the edge effect may be relatively reduced.

### 3.2. Measurements Made on the Human Chest Wall

**Figure 18:**
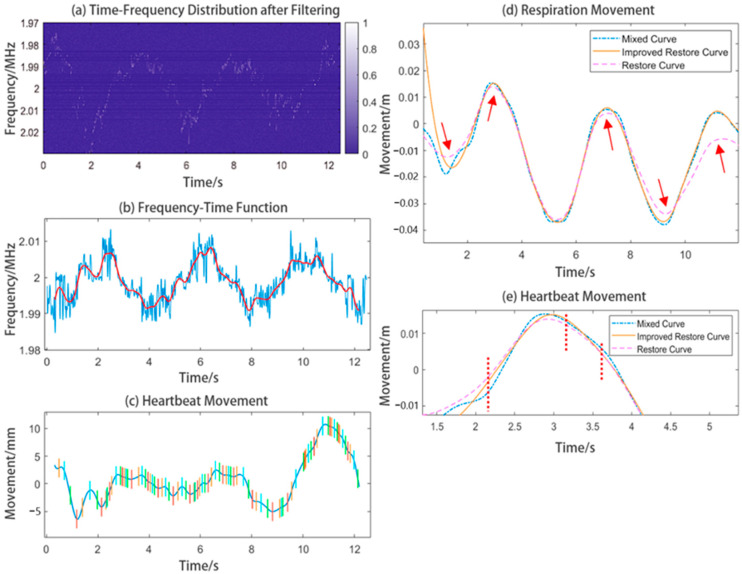
Experiment results of the human chest wall, including the filtered time-frequency graph, centroid curve, and restored respiratory waveform. Some details of the originally restored respiratory waveform and improved respiratory waveform are shown in the figure.

For this measurement, we made the tester sit in the same position as the loudspeaker box with the laser focusing on the chest wall. Although the tester was requested to breathe normally, the influence of the body displacement of the tester on the experimental results had not been reduced completely. After extracting by the method mentioned in Section 2.2.2, we obtained the time–frequency graph shown in Figure 18a, and the centorid curve shown in Figure 18b. The geometry features of the curve were not affected by the extremely low SNR, which means the filtering and extracting methods can deal effectively with the signals with extremly low SNRs.

The restoration of the heartbeat waveform using the traditional EEMD method will not work well because of the presence of low-frequency signals in the waveform. These low-frequency signals make it difficult to locate the position of each peak in the heartbeat waveform and distort the respiratory waveform, but we located almost each peak in the heartbeat waveform using the method in Section 2.2.3, as shown in Figure 18c. We can notice that the heartbeat waveform restored by traditional EEMD method contains a lot of drift. This will make the respiratory waveform distorted. In addition, we overlaid the respiration waveform with the mixed curve (which was extracted from the time–frequency plot containing multiple frequency components). We found that the original respiration waveform did not pass through the mixed curve, which would have resulted in mode mixing to some extent. In Figure 18d, the points in the pink waveform at which arrows are placed are distorted. The original respiratory waveform is too high or too low, such that it bypasses the mixed waveform.

However, by using the waveform fixing method in Section 2.2.3, our improved respiration waveform was fixed adaptively and passed well through the mixed curve. Thus, the respiration waveform can be considered a basic waveform. Its details are shown in Figure 18d, in which we can see that the improved waveform (orange curve) has a better shape than the original waveform (pink curve). Figure 18e shows the details in a certain peak of the mixed waveform. We used a red line in Figure 18e to compare the positions of the original respiratory waveform and the fixed respiratory waveform. We can clearly see that the fixed waveform goes through the mixed waveform, instead of missing the vibration of the curve. In ideal conditions, the heartbeat waveform should be modulated in the form of amplitude on the respiratory waveform. The experimental results indicate that the fixed curve can show more obvious features of “base wave” after being processed by the method in this paper.

**Figure 19:**
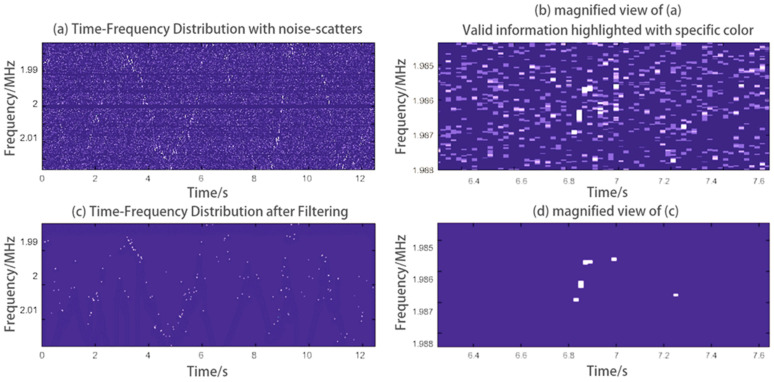
Another experimental result on human chest wall. The original data has extremely low SNR and the time-frequency graph cannot be directly used to extract centroid curve. But by using the filtering method in Section 2.2.2, we obtained an improved graph with more obvious features. For better visual observation, the contrast and saturation of the image have been enhanced.

In Figure 19a shows a time–frequency graph of experimental data with extremely low SNR, and (b) is some details at a certain position in graph (a). We can see the original graph is almost inundated by noise. This noise inteferes with the position and shape of the centroid curve. By using the filtering method mentioned in Section 2.2.2, we can obtain the filtered time–frequency graph shown in (c,d) shows the details at the same position. The graph has a higher SNR.

**Figure 20:**
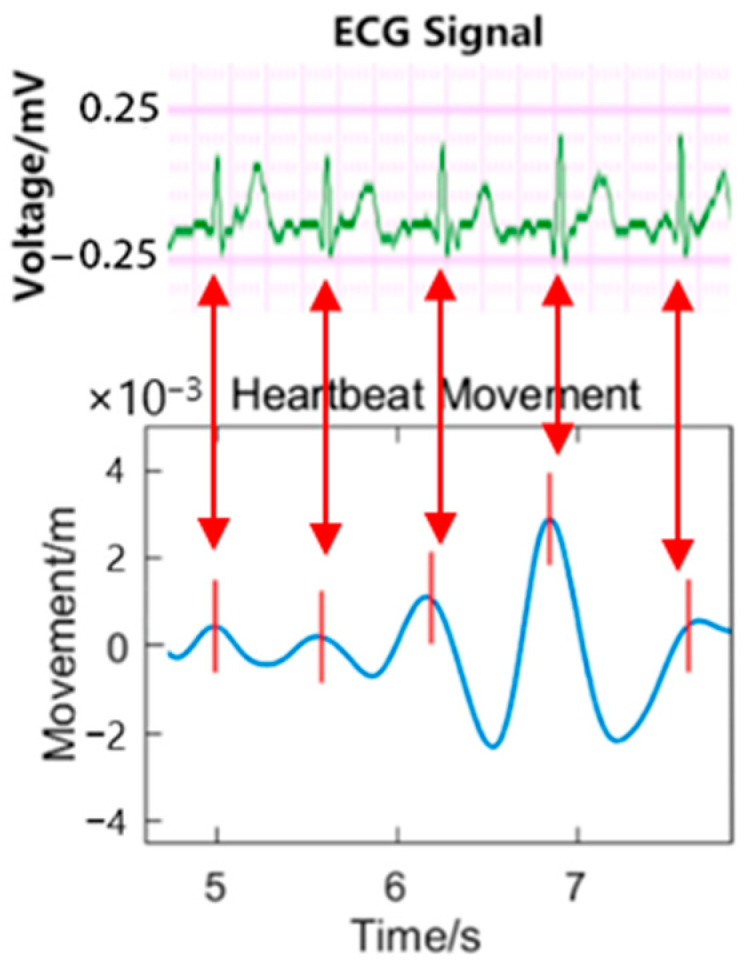
The comparison between ECG signal and heartbeat waveform. The peaks in the heartbeat waveform may not correspond to the R-peaks in ECG signal in the physical sense, but the time gaps are almost the same. Green curve: ECG-measured heartbeat signal; Blue curve: restored heartbeat movement; Red line: extracted peak positions. The red arrows indicate the potential correlations between two curves.

Furthermore, we used the same algorithm to process these experimental data, and obtained the restored heartbeat and respiratory waveform. Figure 20 shows the heartbeat waveform extracted from the graph. In this experiment, we record the ECG signal of the tester simultaneously by using an ECG monitoring device, and obtained the heart rate of the tester. This rate is compared with the rate obtained by the Laser-Doppler system, and the results showed that the minimum relative error between the measurements was 4.2%, and that the heart rate measurement accuracy can reach approximately 95%.

In order to verify the relationship between the heartbeat waveform and the ECG signal, the length of time period of ECG and heartbeat waveform are compared, and the peaks are synchronous with the R-peaks in the ECG signal at some time. Combined with the conclusions of related works [6,23], this result indicates the effectiveness of the algorithm to some extent.

## 4. Conclusions

In this paper, we presented several new algorithms for optimizing the extraction of vital signals of patients based on the Laser-Doppler effect, and demonstrated the validity of the algorithms through experiments. The results of the experiments indicate that the proposed algorithms improve the adaptability and robustness of the measuring system. The ability to extract valid information is enhanced when using the proposed system, particularly in the presence of strong IF interference and low SNR signals. The shape of the restored waveforms can be adaptively corrected based on the results of EEMD. The algorithms enabled the system to make measurements, even in unpredictable environments, reducing the requirement for specific measurement conditions, such as those pertaining to surface reflectivity or body-movement limitations, and they would facilitate further research on decoupling the waveforms with enhanced accuracy.

We noted that the frequency of the high-frequency waveform measured in the chest wall experiment was less accurate than the frequency of the waveform measured in the loudspeaker experiment. This difference in the accuracies of the frequencies can be attributed to low amplitude chest vibrations caused by body movements or other vital movements. Concerning body movements, we can request the tester to stay still during the experiment to reduce the interference. For vital movements other than those due to heartbeat and respiration, further research would be required in which we would also be able to use waveforms to represent body movements. Once the components and regulations have been specified, it will be possible to restore the two waveforms effectively.

The respiratory frequency was not measured during the experiments because of the nonavailability of a portable respiratory monitor. However, from the results of the experiment on the loudspeaker box, we found that the longer the sample time is, the higher the reduction of the edge effect of the low-frequency waveform will be. The relationship between the edge effect and sample time duration requires further investigation.

## Data Availability

The data presented in this study are available on request from the corresponding author. The data are not publicly available due to that datas includes vital signal features of the testers.
